# Survey data on preferences and attitudes towards participatory irrigation management in India and Pakistan

**DOI:** 10.1038/s41597-023-02052-y

**Published:** 2023-03-21

**Authors:** Bethany Cooper, Lin Crase, Michael Burton, Hung Duy Pham

**Affiliations:** 1 School of Business, University of South Australia, Adelaide, Australia; 2 School of Agriculture and Environment, University of Western Australia, Perth, Australia

**Keywords:** Water resources, Developing world, Agriculture

## Abstract

The data described in this paper were collected from four jurisdictions in south Asia, Assam and Bihar in India and Punjab and Sindh in Pakistan. The data were collected from farmer households involved in surface water irrigation with the aim of understanding the merits of participatory irrigation management (PIM) in different settings in south Asia. The data were collected using four structured survey instruments, which comprised three paper-based surveys and one online survey collected via tablets. This data can be used by researchers to empirically analyze: overall institutional performance and its relationship to agro-economic variables; drivers of compliance; gender differences and their impact on participation in water groups and perceptions of performance; preferred charging regimes and broader institutional arrangements for managing water at the local level. These data are unique, having been collected simultaneously across the four jurisdictions.

## Background and Summary

Participatory irrigation management, where farmers assume more responsibility for the administration of surface water in centrally controlled irrigation systems, has been a mainstay of irrigation reforms for at least forty years^1^. Throughout that time participatory irrigation management has been heavily promoted by donor agencies, by tying monetary support for irrigation development and upgrades to a commitment from recipient states to embrace more farmer-led decision making^2^. In many cases there has also been a strong domestic incentive, with national and sub-national governments facing substantial financial burdens for ageing irrigation infrastructure and a motivation to thus shift liability away from the public purse^3^.

Despite the enduring keenness for promoting participatory irrigation management, the approach has yielded only mixed success^4^. A substantial literature has emerged on this topic, including efforts to elucidate the ingredients for more successful practices in specific locations^5,6^. Some cross-jurisdictional comparisons have been drawn, but these invariably attempt to bring together strands of research work performed differently at different locations and/or across different time horizons^1^. Cross-jurisdictional studies undertaken with a similar methodological approach, equivalent data gathering and covering similar time frames are rare.

In both Pakistan and India, the management of irrigation largely falls to sub-national governments (provinces in Pakistan and states in India). In this study, data were drawn simultaneously from farm households covering four jurisdictions in south Asia – two states in India (Assam and Bihar) and two provinces in Pakistan (Punjab and Sindh). Whilst all farmers were part of water user associations (WUAs) the role and functioning of those associations varied as did the amount of decision making that was retained centrally. For example, WUAs in all four jurisdictions are involved in the collection of irrigation fees from farmers but the levies, rate-setting processes, and compliance measures differ. In some cases, almost all monies are returned to the state government irrigation department and in other cases the majority of funds are retained locally and expended by the local WUA. In Assam, all collected fees are expected to be passed to the state while in Bihar 70% is kept locally. In Sindh, 60% of collected fees are passed to government and in Punjab, the collections are halved between local and provincial authorities^7^.

The hierarchical structures that circumscribe WUAs also differ and in some cases nomenclature is unique. In Pakistan, WUAs operate at the watercourse level (or khal panchayat) that then elect a chairperson to sit on farmer organisations that purportedly have responsibility at the distributary level. Representatives from the farmer organisations are then intended to sit on Area Water Boards that operate at the canal command area. Responsibility for developing these organisational structures rests with the Sindh Irrigation and Drainage Authority (SIDA) in the case of Sindh and the Punjab Irrigation and Drainage Authority (PIDA) was formed to accomplish the task in Punjab^8^. In 2019 PIDA was abolished, however the data collected for this study occurred prior to that, providing an opportunity for future comparative analysis against subsequent arrangements.

In India, WUAs operate at the lowest tier and the hierarchical structures vary by state. In Bihar, WUAs are voluntarily formed and constituted using village level committees (VLC). System level committees (SLC) reside at the next level and are made up of representatives from VLC. The Water Resources Department is the superordinate tier. In Assam, WUAs are delineated and declared by the District Collector. In contrast to Bihar, WUAs are managed by a committee that includes farmer and government representatives. Distributary committees (DC) form the next tier and are comprised of presidents of WUAs (to a maximum of 5) and officials from the irrigation and agriculture departments. Project committees (PC) make up the next tier and include the Executive Engineer from the irrigation department and District Agricultural Officers from agriculture. An apex committee is the highest tier and includes the Minister for Irrigation, elected representatives from PCs and state officials^9^.

Of interest was the extent to which these types of institutional characteristics related to different levels of irrigation and agricultural performance.

Given the interest of this work around the influence of institutions on performance, the project had initially planned the collection of data to address two main research topics. The first was to explore how concepts related to New Institutional Economics and governance theory impacted on performance. This component aimed to capture whether a WUA had clear objectives, capacity to bring compliance, was established at a suitable scale, had capacity to adapt and was consistent with local norms and thus encouraged interaction amongst members. In addition, we sought to understand whether a WUA met specific rationalities drawn from governance literature (e.g. technical rationality measures the degree of competence to make relevant decisions about infrastructure; organizational rationality measures the extent to which structures are functioning and can bring financial discipline). The ambition was to empirically track these institutional and governance features against agricultural performance^10^.

The second topic of interest was the capacity of local institutions to bring compliance amongst irrigation farmers. Failure to secure irrigation payments and breaching water access rules is frequently presented as one of the main challenges of participatory irrigation management^11^. To gain insights into this topic, we sought to invoke the theory of planned behavior (TPB) which posits that behavior (in this case compliance) is driven by attitudes, subjective norms and perceived behavioral control^12^. Measuring these constructs against compliance outcomes was the goal.

As the project progressed through the initial case study phase, two additional research topics emerged. First, it was clear that a gender perspective would not be clearly captured within the extant focus and a decision was made to elicit the views of men and women separately to gain an appreciation of differences in perception about performance. This approach has previously shown to reveal important nuances about the roles of gender^13^. This also provided an opportunity to consider concepts related to the empowerment of women^14,15^.

Even though the focus was on existing institutional characteristics and the links to performance, there remained questions about the extent to which farmers would prefer alternative arrangements. This formed the second additional research topic and two separate, but related issues emerged – one dealing with how water charges are derived and collected and another focused on alternative governance arrangements and decision making authority. In this case, discrete choice experiments (DCE) were identified as a useful method to quantify these preferences. DCEs are particularly helpful when a ‘new product or policy’ comprising different attributes is being offered^7^. The first experiment would interrogate preferences for different charging and collection regimes while the second focused on how revenues were controlled and the extent to which responsibilities were solely vested in the WUA versus shared or assigned to the state. A DCE requires a statistical design that established the choice sets faced by respondents. Pre-testing can also be used to generate priors to then modify the design. An initial pre-test of the experiments was undertaken using an orthogonal design. Subsequently, a Bayesian efficient design was employed, based on priors, to structure the experiment on charging regimes. This resulted in 36 choice sets that were blocked into 9 sets of 4 choice questions. In the case of the experiment dealing with broader governance structures, the pre-test resulted in the removal of some attributes and it was possible to invoke a full factorial design of 36 choice sets. The statistical software Ngene was employed to generate the designs and additional detail on this particular DCE can be found in the literature^7^.

Ultimately, tying these different data sets together with common identifiers would allow exploration across a range of issues and thus help explain when and how participatory irrigation management outperformed more centralized approaches.

## Methods

### Sampling approach

Since institutional characteristics and their impact on performance was the main theme, the broader institutional survey covering concepts from New Institutional Economics and governance theories received most attention. The ambition was to assemble sufficient data to analyze pooled data but to also extract insights at the state or provincial level, if required. The nature of some of the data (e.g. Likert measures) and the intention to generate factors from these data meant that sample size needed to be considered. Whilst there is some conjecture in the literature on this topic a sample of approximately 250 per jurisdiction was adjudged sufficient^16^.

The breadth of the institutional survey and the related cognitive burdens on participants meant that it was impractical to have every respondent address all four research topics. Accordingly, sub-samples were chosen from the participants in the overall institutional survey to then complete questions relating to compliance, gender differences and preferences for new institutions, respectively. In this case, a sample of 50 from each jurisdiction was deemed sufficient to gain insights with a total pooled sub-sample of 200 for each of these research topics. In the case of the gender survey, female enumerators were employed to administer surveys to female participants to provide a point of comparison with some of the data in the institutional survey completed predominantly by male household heads.

As noted, the four jurisdictions of interest exhibited different rules around formal WUA design so a sample covering all jurisdictions was required. It was also plausible that WUAs would vary in their performance within jurisdictions for a variety of reasons^17^. State and provincial government officials were thus asked to identify instances where WUAs had performed differently in order to capture some of this variability. In India, this screening was defined at the Central Level Committees and in Pakistan Area Water Boards were used as the reference level for performance. Other studies have also shown that irrigation and farm performance can be impacted by the location of farms within the irrigation network, with farms located towards the head of the network generally outperforming those in the middle or tail of the network^18^. As a result, identified districts were then overlaid with the area of the irrigation distribution network to ensure coverage of farmers at the head, middle and tail of the irrigation scheme.

**Fig. 1:**
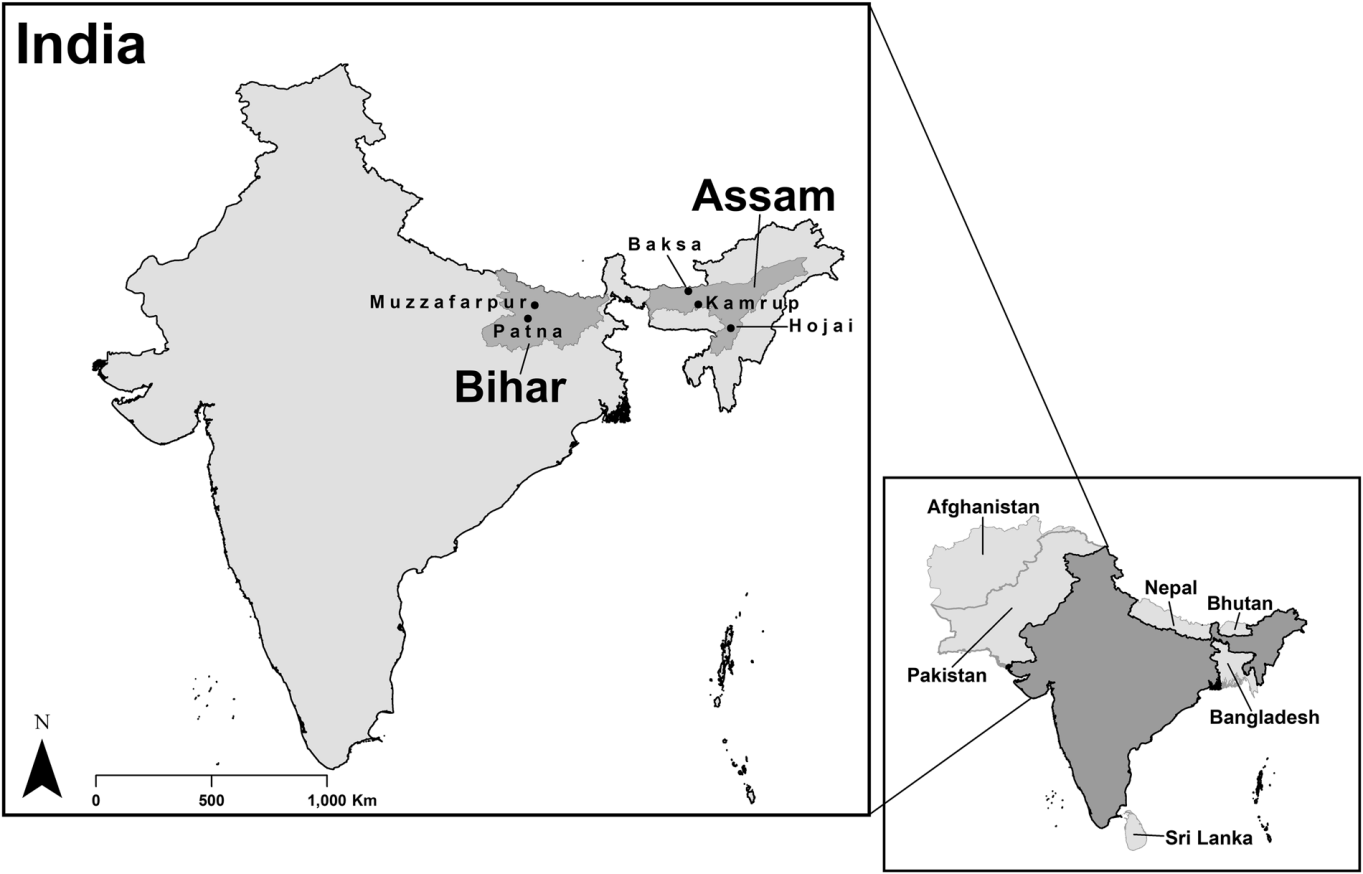
Map of India: Study location States (GADM data underpins the map https://gadm.org/).

**Fig. 2:**
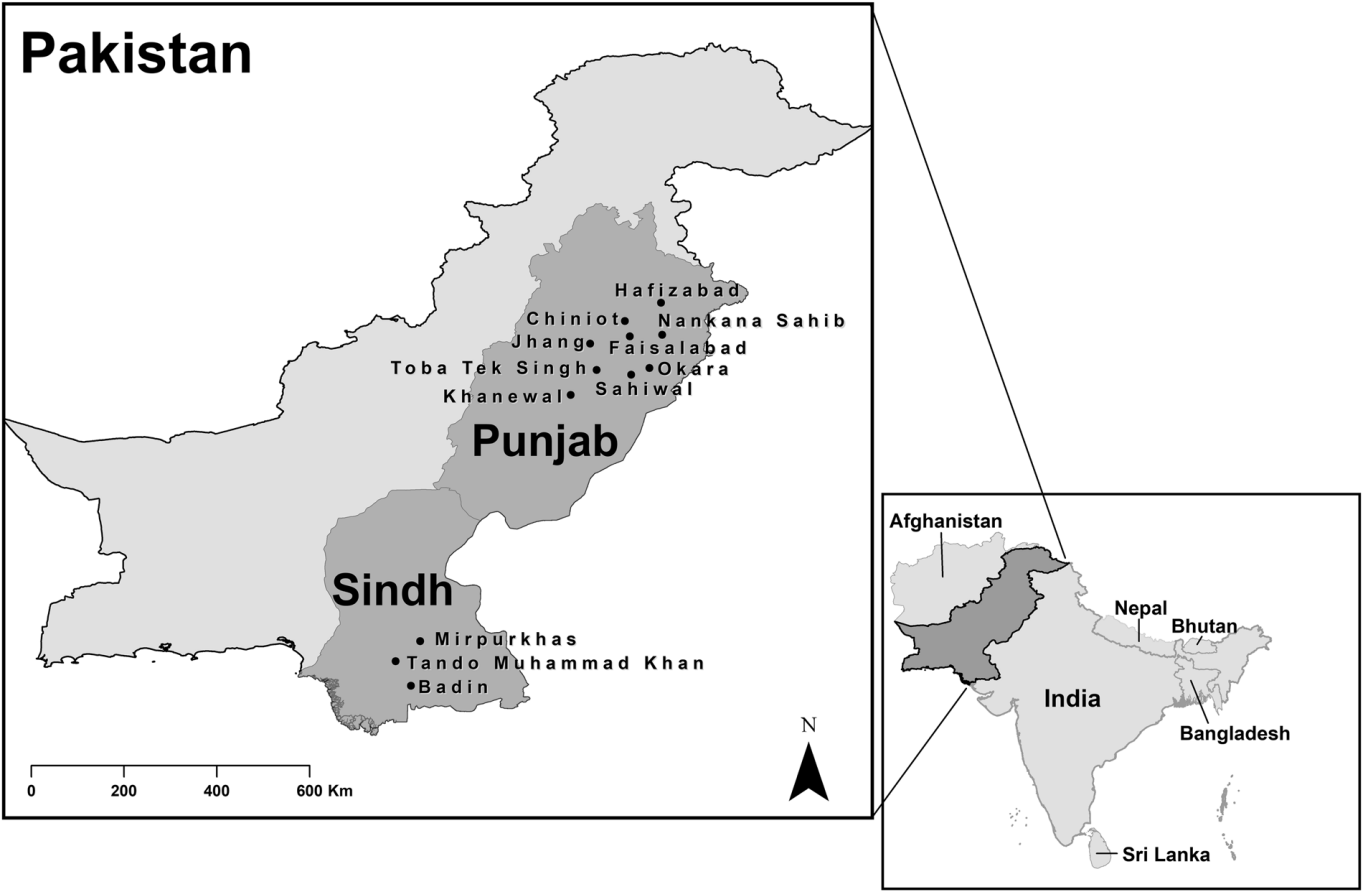
Map of Pakistan: Study location Districts (GADM data underpins the map https://gadm.org/).

The selected districts in Assam were Kamrup, Hojai, and Baksa and in Bihar the districts were Patna and Muzzafarpur (see, Fig. 1). Approximate quotas were then assigned to gain coverage from across the different districts while achieving the desired sample size. From within the districts in Assam, 19 villages were identified that covered 25 village level committees and gave the spread across the irrigation network. In Bihar 26 villages were identified that met the sampling requirements. The sampling process in Pakistan was similar. The Punjab districts comprised Hafizabad, Faisalabad, Toba Tek Singh, Nankana Sahib, Chiniot, Jhang, Okara, Sahiwal, and Khanewal (see, Fig. 2) and the districts in Sindh included Tando Muhammad Khan, Badin, Mirpurkhas (both Digri and Shujaabad taluka) (see, Fig. 2). Again, quotas were broadly assigned to gain coverage across the network, although some oversampling arose in Punjab. Within each sampling area households involved in surface water irrigation were randomly invited to participate with enumerators approaching adjacent households in the village if participation was declined.

### Data collection

The enumerators employed for data collection in the two Indian states were recruited and trained by the Indian Institute of Management (Ahmedabad). Mehran University of Engineering and Technology (Jamshoro) recruited and trained enumerators for data collection in Sindh and the University of Agriculture (Faisalabad) was responsible for the enumerators employed for the Punjab data collection. Training ensured that enumerators understood the research questions and how to conduct the interview including data entry for the paper based as well as the tablet surveys. All enumerators were fluent in English as well as the local language of their data collection site. The survey tools used to collect the data were deemed to meet the ethics requirements of the Indian Institute of Management (Ahmedabad), Mehran University of Engineering and Technology (Jamshoro), and the University of Agriculture (Faisalabad) before the study commenced. The surveys dealing with institutional performance, compliance and gender were completed in paper form while the DCE data were collected using tablets. This was done so that the choice sets presented to respondents could automatically populate and to ensure that the choice sets rotated systematically to give coverage across all choice tasks. The tablet based surveys commenced with a series of questions about the existing charges and administrative arrangements. The responses to these questions auto populated the status quo for the choice sets. In the initial part of the survey, respondents were also asked to rate the extent to which they understood the various rules and the degree to which they and their neighbors complied with the rules. The choice experiment data was collected using a proprietary software application (CommCare) and their data servers. Data was later downloaded from the servers and exported into excel spreadsheets for analysis.

The resulting samples by research topic and jurisdiction are summarized in Table 1.

**Table 1:** Sample by research topic and jurisdiction.

Research Topic	Assam	Bihar	Punjab	Sindh
Institutional performance	252	258	318	250
Compliance	52	63	68	50
Gender	52	57	82	50
Preference (DCE)	198	196	252	173

## Data Records

All files and data are stored in the repository Figshare^19^ and the license type for reuse of the data is CC-BY. The four surveys were administered in India during April-May 2018 and April-July 2018 in Pakistan. Since the nomenclature of some institutions differs across states/provinces, a standardized approach is used in each country. In India the lowest level of WUA is designated as ‘VLC’ (village level committee) and the next highest tier is ‘CLC’ (central level committee). In Pakistan the lowest tier is designated as ‘KP’ (khal panchayat) while the next superordinate tier is recorded as ‘FO’ (farmer organization). Responses to the institutional survey are captured in Excel (xlsx) spreadsheet form. The institutional data covers 12 main areas. The data for India and Pakistan are recorded separately. A broad summary of each data topic and record type appears as Table 2.

**Table 2:** Data summary of institutions survey.

Topic	Data type
Identifier information	Date of collection, collector identifier, type of irrigation system, size of farm, location of village
Profile of respondent	Relationship in household, socio-economic indicators, status in village, status in water institution, location in water command, agricultural land use
Hydrological profile	Sources of irrigation, adequacy of supply, water scarcity, changes in water table depth, changes in availability and institutional environment
Water institution	Structural nature and working of local water institution, involvement in and inclusiveness of institution, extent of devolved decision making
Assessment of rationalities in context of water institution	Likert scale items covering technical, environmental, economic, social, political, organizational, financial, government rationalities
Assessment of institutional characteristics	Likert scale items covering clarity of objectives, coherence between norms, adaptiveness, scale, compliance
Financial performance of farm household	Likert scales measuring crop production, off-farm income, production costs
Long term impact of water institution	Likert scales measuring perceptions of impact on water availability, equity, environment, finance
Other impacts of water institution	Likert scale measures of direct and indirect impacts, major challenges facing water institution
Water rule design and adherence	Likert scale measures of rules related to water distribution, representation, maintenance, payment of fees, role of government
Performance assessment	Likert scale measure of global performance of water institution and performance along water availably/distribution, environmental, economic, equity, financial criteria
Perceptions of success	Open-ended response format for questions covering water institution’s strengths, weaknesses, areas for improvement

The compliance survey data covers 5 topics and again is separated in different xlsx files for the two countries (Pakistan and India). Table 3 provides a summary of each data area and record type.

**Table 3:** Data summary of compliance survey.

Topic	Data type
Identifier information	Date of collection, collector identifier, location of village
Existing rules	Dichotomous responses to the current format of rules, namely: water distribution, collection of fees, transfer of fees to government, repairs and routine maintenance, special repairs, extraordinary capital outlays, election of officials, participation of members
Compliance behavior	Likert scale responses targeting social, moral and economic drivers for compliance across the various rule sets (i.e. water distribution, collection of fees, transfer of fees to government, repairs and routine maintenance, special repairs, extraordinary capital outlays, election of officials, participation of members)
Motivations for compliance	Likert scale responses to overall motivations to comply covering social norms, motivation to comply to social norms, perceived behavioral control, personal attitudes, personal moral values, personal efficacy (effect-results), behavioral intent and formal enforcement
Mechanisms for improving compliance	Dichotomous response to three alternative approaches

Table 4 summarizes the data topics and record for the gender survey, noting these are recorded separately for each country.

**Table 4:** Data summary of gender survey.

Topic	Data type
Identifier information	Date of collection, collector identifier, location of village, survey setting (e.g. woman alone or accompanied)
Profile of respondent	Relationship in household, socio-economic indicators, status in village, agricultural land use
Structural elements of water institution and women	Dichotomous responses comprising: respondent’s own engagement with institution, respondent’s understanding of structure of water institution, involvement of other women. Likert scale items (1–5 response format) comprising: barriers to involvement of women, benefits of women’s involvement
Involvement of women in household decisions and water/irrigation	Categorial response comprising decision made by man, woman or jointly in case of own household, followed by Likert scale response to women’s involvement in decision domain generally
Leadership and community influence	Dichotomous and Likert scale responses covering women’s comfort speaking in public, women’s involvement in other groups/institutions
Assessment of rationalities in context of water institution	Likert scale items covering technical, environmental, economic, social, political, organizational, financial, government rationalities
Assessment of institutional characteristics	Likert scale items covering clarity of objectives, coherence between norms, adaptiveness, scale, compliance
Long term impact of water institution	Likert scales measuring perceptions of impact on water availability, equity, environment, finance
Other impacts of water institution	Likert scale measures of direct and indirect impacts, major challenges facing water institution
Water rule design for inclusion	Likert scale measures of rules related to involvement of women
Performance assessment	Likert scale measure of global performance of water institution and performance along water availably/distribution, environmental, economic, equity, financial criteria
Perceptions of success	Open-ended response format for questions covering water institution’s strengths, weaknesses, areas for improvement

In the case of the choice (DCE) data, surveys were administered electronically but Internet connectivity was not always available at the time of interview. Accordingly, data were uploaded each evening. The data record for each state/province is held separately as CSV files; resulting in 8 data files in total. Note that the data for these surveys is reported in ‘long’ format, with one line in the file for each alternative, for each of the choice sets viewed by the respondents. The attribute levels for each alternative seen by the respondent are also reported. The choice scenarios, attributes and related levels are described in full in Burton, Cooper and Crase^7^. The structure of the data files is summarized in Table 5.

**Table 5:** Data summary of DCE survey.

Topic	Data type
Identifier information	Respondent identifier linked to institutional survey
Choice set details and response – 2 separate experiments dealing with charging regimes and decision-making structures, respectively.	Choice block and set within block and respondent’s choice from set (1 = selected, 0 = not selected), and the attribute levels within each option.
Fee payment	Likert scale response to frequency of payment by self and likely payment by neighbors, and monetary value of fees currently charged by water institution
Protestor responses	A series of questions identifying if respondents were protesting about the choice experiment rather than choosing an alternative
Understanding of respondent	Likert scale responses to the extent the respondent felt they understood the questions and had sufficient information
Cheap talk	Likert scale responses to the extent to which respondent thinks their choices make a difference and would result in them acting on those responses
Assessment of knowledge of institutions rules	Likert scale response rating respondent’s own knowledge of institutions rules

## Technical Validation

Pre-testing of the survey instruments occurred in India, primarily because monsoon rainfall arrives earlier in the two eastern states of India and field survey administration become impractical post-monsoon. The pre-testing of the DCE survey indicated that the number of choice tasks per respondent needed to be reduced due to the cognitive burden. The decision to auto-populate the status quo on site for the choice sets in the DCE also derived from this pre-test phase when it became obvious that the local interpretation and application of rules differed from the published information at the state/provincial level. The daily uploading of the DCE data allowed for simultaneous preliminary modelling.

Responses to the paper based surveys were initially entered into Excel spreadsheets by enumerators in each country using common entry instructions. The resulting spreadsheets were assembled for cleansing by a single administrator in Australia. The administrator systematically identified anomalies across the different data sets and liaised with the survey teams in south Asia to resolve inconsistencies. This was primarily around ensuring that individual respondents’ common identifiers had been recorded correctly across the different survey instruments.

In some cases, data cells have not been populated. This has occurred because not all questions were applicable across the different jurisdictions. For example, in Pakistan questions around caste are redundant given the cultural differences between the two countries. In addition, several qualifying questions make subsequent questions superfluous. Intentional blanks include the indicator of −9999.

## Usage Notes

Data have been deidentified and standardized across the different data files. The data collected on institutions, gender and compliance are in formats that can be interrogated by any statistical program capable of performing principal component analysis and structural equation modelling. Several examples of applications appear in the literature^7–11,14,15^. There is scope to use more simplified analytical tools.

The choice data can be modelled using a range of statistical software packages (e.g. Stata, Nlogit, R). Earlier attempts to pool these data across the four states/provinces have not proven useful, although analysis of preferences, while interacting other considerations (e.g., current compliance with fee payment) has shown some promise^7^.

The data files related to the institutions, compliance, gender and DCE survey files can be accessed via 10.6084/m9.figshare.22216828^19^.

## Data Availability

No code was used to generate or process the current dataset.
